# Monoclonal Antibodies in Smoldering Multiple Myeloma and Monoclonal Gammopathy of Undetermined Significance: Current Status and Future Directions

**DOI:** 10.3390/ph17070901

**Published:** 2024-07-06

**Authors:** Valeria Ferla, Francesca Farina, Tommaso Perini, Magda Marcatti, Fabio Ciceri

**Affiliations:** 1 Hematology and Bone Marrow Transplantation, IRCCS San Raffaele Scientific Institute, 20132 Milan, Italy; farina.francesca@hsr.it (F.F.); perini.tommaso@hsr.it (T.P.); marcatti.magda@hsr.it (M.M.); ciceri.fabio@hsr.it (F.C.); 2 Age Related Diseases Laboratory, Division of Genetics and Cell Biology, IRCSS San Raffaele Scientific Institute, 20132 Milan, Italy; 3 Faculty of Medicine and Surgery, Vita-Salute IRCCS San Raffaele University, 20132 Milan, Italy

**Keywords:** smoldering multiple myeloma, monoclonal gammopathy of undetermined significance, monoclonal antibodies, therapy, prognosis

## Abstract

Monoclonal antibodies (MoAbs) targeting several cellular receptors have significantly improved the prognosis of multiple myeloma (MM). Their high effectiveness and safety raise the question of whether earlier therapeutic intervention in monoclonal gammopathy of undetermined significance (MGUS) and smoldering multiple myeloma (SMM) influences the natural course of the disease. MM is preceded by clinically recognized conditions such as MGUS and SMM. Numerous studies are investigating the disease biology and immune profile of SMM and MGUS to unravel the intricate relationship between immunosurveillance and disease progression. The standard approach to MGUS and SMM remains close observation. Early studies indicate benefits in terms of progression or even survival for promptly treating high-risk SMM patients. Ongoing debates are focused on which patients with SMM and MGUS to treat, as well as on determining the optimal therapeutic approach. The first approach aims to cure by attempting to eliminate the pathological clone, while the second approach is preventive, aiming to manage disease progression to active MM and restore the immune system. In this review, we focus on the available and emerging data on early treatment, particularly with MoAbs alone or in combination with other therapies, in SMM and MGUS patients.

## 1. Introduction

In recent years, the landscape of multiple myeloma therapy has been transformed by the introduction of novel classes of drugs, providing benefits to both newly diagnosed and relapsed/refractory patients [1]. These advancements, particularly the incorporation of monoclonal antibodies targeting various cellular receptors, for example, CD-38 and SLAM F7, in combination with other classes of agents, have substantially improved patient outcomes across different disease stages [2,3,4]. Furthermore, the transition from triplets to quadruplets incorporating MoAbs has not resulted in a decrease in patients’ quality of life, attributable to their mechanism of action and safety profile [2,3].

Smoldering Multiple Myeloma is an asymptomatic stage between MGUS and active MM [5]. Traditionally, the standard approach has been observation until the occurrence of a myeloma-defining event (MDE) [6]. However, early introduction of lenalidomide, either alone or with dexamethasone, in two pivotal trials showed a significant improvement in time to progression and prevention of organ damage in high-risk SMM. Notably, one of these studies indicated an overall survival benefit [7,8]. Based on these findings, phase 2 and phase 3 studies have been initiated with various pharmacological agents, both as single agents and in combination, with the aim of optimizing the results obtained with immunomodulatory agents. The optimal therapeutic approach, whether the “curative” or the “preventive” approach, remains a subject of investigation [9].

In this review, we present the attained results and ongoing trials, with particular emphasis on MoAbs in SMM and MGUS. We aim to highlight uncertainties and critical issues that must be resolved before the “watch and wait” paradigm can be replaced with early treatment.

## 2. Epidemiology and Clinical Presentation

Clonal plasma cell proliferative disorders include MGUS, SMM, and MM. The vast majority of patients with MM progress from MGUS, a precancerous stage. MGUS is relatively common, detected in approximately 5% of adults over age 50, although its incidence varies by ethnicity [10,11,12,13,14]. Long-term follow-up studies have shown that MGUS progresses to MM at a rate of 1% per year [15,16].

Prevalence estimates for MGUS have been based on testing using serum protein electrophoresis and serum immunofixation, which are the primary methods for identifying the presence of a monoclonal component. The serum-free light-chain assay is employed to determine the monoclonal light-chain component [12,13]. Techniques such as mass spectrometry, including Matrix-Assisted Laser Desorption/Ionization-Time of Flight (MALDI-TOF) and Electrospray-Ionization Time-of-Flight Mass Spectrometry (miRAMM), have demonstrated greater sensitivity [17,18]. These advanced techniques can detect monoclonal components that are not identifiable by serum electrophoresis and immunofixation, reducing the number of false positives at diagnosis. They utilize immunoglobulin-targeted nanobodies, aiding in the identification and quantification of monoclonal proteins. Moreover, mass spectrometric assays allow for accurate follow-up of the identified monoclonal component, as the molecular weight of the M-protein light chain is a specific and reliable marker of the plasma cell clone. This method offers multiple advantages, including cost-effectiveness, reduced processing time, and high automation [19]. In the future, mass spectrometry could potentially replace serum immunofixation, providing greater accuracy in detecting gammopathies and eliminating false-positive cases.

A rare condition called monoclonal gammopathy of clinical significance (MGCS) differs from MGUS/SMM due to specific clinical manifestations and organ paraprotein-related organ damage. In these patients, early diagnosis is essential to promptly start a treatment that preserves the functionality of the organ. In particular, monoclonal gammopathy of renal significance (MGRS) produces light and/or heavy nephrotoxic monoclonal chain immunoglobulins [20].

SMM affects approximately 0.5% of the population over the age of 40 [21] and accounts for about 15% of new MM diagnoses [22,23,24]. From a prognostic point of view, the distinction between SMM and MGUS is crucial, given the higher risk of progression to symptomatic MM. Specifically, SMM carries a risk of progression of approximately 10% in the first five years, 3% in the following five years, and subsequently decreases to 1.5% per year. However, it is important to note that SMM is a highly heterogeneous clinical condition and we currently do not have adequate biochemical and molecular tools to better stratify its behavior. Approximately two-thirds of patients clinically resemble MGUS, while in other cases, it is more similar to MM. Furthermore, the decreased risk of progression to symptomatic MM in the years following diagnosis suggests that SMM is not simply an intermediate stage between MGUS and MM [5,25,26].

For a long time, the diagnosis of MM required evidence of end-organ damage, known as the CRAB features, which includes hypercalcemia, renal failure, anemia, and bone lesions. In 2014, the International Myeloma Working Group (IMWG) modified these criteria by adding three myeloma-defining events, also called SLiM CRAB criteria: the presence of at least 60% clonal plasma cells in the bone marrow (BMPC), an involved/uninvolved serum free light chain ratio (FLC) equal to or greater than 100 and presence of one or more focal lesions on magnetic resonance imaging (MRI) measuring at least 5 mm. The presence of any of these three markers is sufficient for the diagnosis of MM. Each of these three markers carries a greater than 80% risk of progressing to symptomatic myeloma within two years, according to CRAB criteria. This group of patients has been defined as high-risk SMM, and it is indicated to start therapy before end-organ damage occurs [5].

## 3. Prognosis and Risk Stratification

Over the years, various attempts have been made to develop risk stratification models by combining different prognostic factors to identify the risk of progression in patients with MGUS and SMM. These are summarized in Table 1 and Table 2, respectively. There are no studies comparing these prognostic scores with each other.

Specifically, the Mayo Clinic identified three risk factors in MGUS: serum monoclonal (M) protein level >15 g/L, non-IgG isotype (IgA or IgM), and serum FLC ratio <0.26 or >1.6. Based on the number of risk factors present, four risk classes were identified: low risk (0 factors), low–intermediate (1 risk factor), high–intermediate (2 risk factors), and high risk (3 risk factors). These categories have an absolute 20-year risk of progression of 5% for the low-risk category, 21% for the low–intermediate category, 37% for the high–intermediate category, and 58% for the high-risk category [27,35].

Also, there are various risk scores in SMM, as shown in Table 2. Currently, the optimal objective of a score is to identify 50% of patients who progress over two years [26,29,31,32,36,37,38,39,40].

Mayo 2018 identified three variables, also referred to as the 20-20-20 score, that enable the identification of patients with high-risk SMM: serum FLC ratio > 20, serum M protein level > 2 gm/dL, and bone marrow clonal plasma cells > 20% [33]. The absence of these factors indicates a low-risk class; one factor indicates an intermediate-risk class and two or more factors indicate a high-risk class. These risk classes have respective progression times of 110 months, 68 months, and 29 months.

The Mayo 2018 criteria have been validated in a second cohort by IMWG and maintain their prognostic value dynamically over the course of follow-up. Increases in serum M protein, FLC ratio, or bone marrow plasma cells can change the risk class throughout follow-up. The IMWG has further refined a more precise score by incorporating additional variables: serum M-protein concentration, FLC ratio, BMPC, and the presence of cytogenetic abnormalities detected via fluorescent in situ hybridization (FISH), such as t(4;14), t(14;16), del(13q), and amp(1q), resulting in a four-group risk stratification model. The two-year progression rates are 3.8%, 26.2%, 51.1%, and 72.5% for the low, intermediate-low, intermediate, and high-risk groups [34].

## 4. Pathogenesis and Immune Regulation

SMM shares recurrent alterations with MGUS, such as multiple trisomies of odd chromosomes or translocations of oncogenes in the immunoglobulin heavy chain (IGH) locus on chromosome 14. These alterations are insufficient for progression toward MM; the progression is characterized by a continuous acquisition of further genomic events, each with distinct behavior. High-risk SMM presents a genomic landscape more similar to that of MM. Abnormalities such as del(17p), del(13q), and amp(1q) increase in frequency with progression to active disease, along with translocations between the IGH locus and the MYC oncogene and the appearance of complex rearrangements [41,42].

Furthermore, impaired immune cell function characterized the bone marrow (BM) microenvironment of MM. Alterations in the bone marrow microenvironment are crucial in determining the indolent behavior of MGUS/SMM or the progression to symptomatic forms [43]. Both intrinsic factors within tumor cells and extrinsic factors, related to the interaction of clonal plasma cells with other immune cells, stromal cells, endothelial cells, and bone cells, play a significant role in disease control. When the disease progresses towards a symptomatic form, there is a loss of the normal bone marrow microenvironment and, therefore, an alteration of the immune control of the malignant clone. In particular, the loss of effector function of natural killer cells and T cells is associated with tumor evasion from immune surveillance [7,26,44].

Understanding the role of immune regulation provides a rationale for immunotherapy, a strategy based on activating the immune system to target myeloma cells. Indeed, monoclonal antibodies that target specific tumor antigens and reverse the immunosuppressive BM microenvironment have shown great promise [45].

## 5. Initial Study on Early Treatment Approach

**Figure 1:**
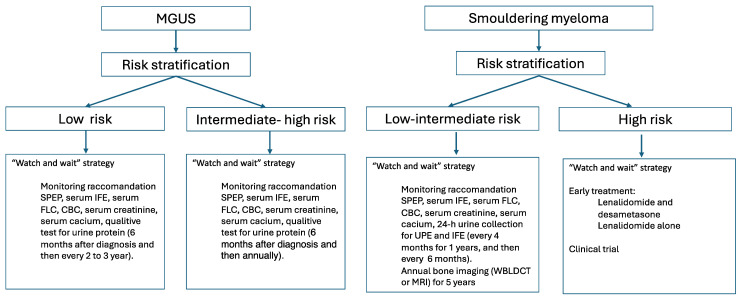
Management of MGUS and SMM. CBC: complete blood count; IFE: immunofixation; FLC: free light chain; SPEP: serum protein electrophoresis; UPE: urine proteine electrophoresis; WBLDCT, whole body low dose CT; MRI: Magnetic resonance imaging.

The guidelines for MGUS and SMM recommend active periodic observation until the appearance of MDE or enrollment in clinical trials (Figure 1) [46,47,48,49,50,51].

In SMM, alkylating agents were found to provide no significant benefit in early treatment [52,53,54]. Another trial comparing zoledronic acid alone to thalidomide plus zoledronic acid showed some benefits in terms of controlling monoclonal components but without any benefit in overall survival or in preventing end-organ damage [55]. Additionally, thalidomide is associated with long-term side effects that render it unsuitable for asymptomatic SMM patients [56,57].

Pamidronate, administered monthly for one year in a randomized trial, demonstrated a reduction in skeletal-related events (SRE) compared to observation alone. However, it showed no advantage regarding time to progression (TTP) or overall survival OS [58]. Similarly, another randomized study highlighted how monthly administration of zoledronic acid for one year reduces SREs [59]. The lack of good risk-stratification models limited these trials.

Two randomized trials have shown that lenalidomide provides benefits in high-risk SMM. In these patients, its immunomodulatory activity seems to activate the impaired immune system [43].

The first study that paved the way was that of the Spanish group (PETHEMA-GEM). A randomized trial (QuiReDex) in which lenalidomide-dexamethasone (Rd) was compared with observation in 119 high-risk SMM defined on the basis of BMPC > 10% and monoclonal protein > 3 g/dL or, if only one criterion present, BMPC with aberrant phenotype > 95% plus immunoparesis. It demonstrated a significantly longer TTP and OS (median TTP: 9 vs. 2.1 years; median OS: not reached vs. 7.8 years). Time to progression to symptomatic disease in both arms was defined from the date of randomization to the date of the first assessment showing symptomatic disease, which was defined as the development of any of the following: hypercalcemia, bone lesions, renal failure, or anemia. There is an imbalance generated by the different criteria for initiating treatment in the observational arm versus the experimental arm. Infections were the most common nonhematologic adverse events, but they were mainly grade 1 and 2 in severity. More secondary malignant neoplasms (SMP) were reported in the Rd group (6% vs. 2%), although the cumulative risk of an SMP was only a trend towards significance. Early therapy with lenalidomide appears to have no consequences on the quality of response and survival to the next line of therapy, suggesting that the therapy does not select resistant clones [7,60,61]. The Spanish study has some limitations. For instance, patients included in the study may have already met the criteria for reclassification as symptomatic myeloma according to the new IMWG 2014 criteria. Moreover, patients in the observation arm, upon progression to symptomatic myeloma, received a first-line treatment outside the study that was different from lenalidomide and dexamethasone, as this regimen was not approved for first-line treatment at the time.

The Eastern Cooperative Oncology Group (ECOG) randomized trial (E3A06) found that lenalidomide monotherapy, compared to observation, prolongs time to symptomatic MM with end-organ damage in 182 patients with high-risk SMM defined by BMPC > 10% and FLC ratio < 0.26 or >1.65 [8,62]. The most significant benefit from early treatment with lenalidomide was found in patients with high-risk SMM, according to both 2008 and 2018 Mayo Clinic risk models. Moreover, compared to observation, there was no alteration in quality of life in patients who received early treatment. Despite the advantage of PFS, only a few patients achieve deep responses (more than very good partial remission (VGPR)), suggesting that achieving deep responses is optional to achieving prolonged disease control in SMM. Hematological toxicities were as expected, while non-hematological grade 3 or 4 adverse events occurred in 28% of patients. The discontinuation rate was notably high at 50%, with 40% of these cases attributed to adverse events. SPMs were observed in 5.2% of treated patients compared to. 3.5% of untreated patients.

Based on the results of these two trials, some authors suggest starting early treatment with lenalidomide alone or in combination with dexamethasone for two years in high-risk SMM. The decision between using lenalidomide alone or in combination must be tailored to each patient, considering factors such as age and comorbidities. For younger patients, it is recommended to collect peripheral blood stem cells for cryopreservation after approximately 4–6 cycles of lenalidomide [24,51,63].

Currently, based on current evidence, no authors suggest early treatment outside of clinical trials.

On the other hand, patients with MGCS have evidence of organ damage, necessitating early and prompt treatment to preserve renal function and prevent end-stage kidney disease (ESKD) with its significant morbidity and mortality. Guidelines for MGCS are primarily based on anecdotal evidence due to their rarity and the lack of available randomized trial data in MGRS. In this contest, the proteasome inhibitor bortezomib is the most important drug in the treatment of MGRS associated with a plasma cell clone. It has a non-renal metabolism and is typically administered in combination with dexamethasone. Other proteasome inhibitors are currently under investigation [64,65].

## 6. Monoclonal Antibodies and Future Directions

Besides lenalidomide, other myeloma agents are also being tested in SMM and even in MGUS, including immunotherapy with monoclonal antibodies as single agents or in associations with more intensive combination regimens with curative intent [66,67,68]. The strong rational of these trials stands on the ability of immunotherapy with monoclonal antibodies not only to interfere with tumor growth but also to induce changes in the body’s immune system and favor anti-cancer immunity (Table 3).

### 6.1. Monoclonal Antibodies in High-Risk SMM

#### 6.1.1. Anti-CD-38 Monoclonal Antibodies

Daratumumab (Dara) is a monoclonal antibody against the surface receptor CD38, expressed not only on plasma cells but also on natural killer (NK) cells and monocytes. It has an important immunomodulatory action, inducing an activation of NK cells and monocytes and an increase in T cell costimulatory molecules, thus improving the activity of anti-MM phagocytosis ex vivo and in vivo. The immunomodulatory action of Dara makes its use in SMM very promising [45].

The phase 2 randomized CENTAURUS study (NCT02316106) assessed the efficacy of Dara in delaying progression to MM in intermediate/high-risk SMM compared to active monitoring. Three incremental dosing schedules of daratumumab at 16 mg/kg administered intravenous (IV) were evaluated: short (1 cycle, 8 Dara administrations), intermediate (20 cycles, 26 Dara administrations), and intense (20 cycles, 32 Dara administrations). Each cycle lasted 8 weeks. The main endpoint was to achieve a rate of complete response (CR) or better, with an established cut-off of 15%. However, the study did not meet this primary endpoint, as CR rates were far below the threshold in all treatment arms. At a median follow-up of 85.2 months, the ORR and ≥CR rates were higher in the intense and intermediate arms compared to the short arm. The 24-month progression-free survival (PFS) rates were 89.9%, 82.0%, and 75.3%, respectively, indicating the greater efficacy of a prolonged schedule. Median OS was not reached in any arm; however, the 84-month OS rates were 81.3%, 89.5%, and 88.1% in the intense, intermediate, and short arms, respectively. After a median follow-up of 7 years, these findings from this final analysis of CENTAURUS demonstrate the clinical activity of Dara monotherapy in intermediate or high-risk SMM. Dara proved effective in controlling the plasma cell clone even without eradicating it. No new safety concerns occurred. Treatment-emergent adverse events (TEAEs) of grade 3 or 4 occurred in 65.9%, 41.5%, and 15.0% of patients in the intense, intermediate, and short arms, respectively. The most common grade 3 or 4 TEAEs were hyperglycemia and hypertension. TEAEs leading to treatment discontinuation were observed in 7.3%, 2.4%, and 5.0% of patients in the intense, intermediate, and short arms, respectively [67,69,80].

Based on the clinical findings from this phase 2 trial, a subsequent phase 3 trial called AQUILA (NCT03301220) has been designed. Its aim is to evaluate the subcutaneous (SC) administration of Dara compared to observation in individuals with high-Risk SMM defined by Mayo Criteria. Dara is administered weekly during cycles 1 and 2, every 2 weeks in cycles 3–6, and subsequently every 4 weeks until completion of 39 cycles (28 days/cycle), up to 36 months, or until disease progression occurs. The primary end point of this study is progression-free survival (PFS). It has completed accrual and is awaiting analysis [70].

Taking into account the distinct mechanisms of action of immunotherapy and chemotherapy, such as dexamethasone and lenalidomide, in inhibiting the growth and spread of tumor cells, a randomized phase 3 trial (DETER-SMM, NCT03937635) was designed to evaluate if a triple regimen, similar to those used in the treatment of MM, may outperform lenalidomide in the treatment of high-risk SMM. This study compares lenalidomide (orally every day, days 1–21) and dexamethasone (orally on days 1, 8, 15, and 22 in cycles 1–12) versus daratumumab (IV on days 1, 8, 15 and 22 of cycles 1–2, days 1 and 15 of cycles 3–6 and day 1 of cycles 7–24), lenalidomide and dexamethasone. The duration of each cycle is 28 days for a maximum of 24 cycles unless there is disease progression or intolerable toxicity. The primary outcome measure is overall survival.

A phase 2 single-arm study (B-PRISM, NCT04775550) is evaluating the efficacy of the quadruplet regimen consisting of daratumumab, lenalidomide, bortezomib, and dexamethasone (D-RVD) in treating high-risk SMM and preventing progression to active multiple myeloma. Daratumumab is administered SC according to standard dose and schedule, while bortezomib is given weekly on days 1, 8, and 15 for cycles 1–6 and then biweekly. Lenalidomide is administered on days 1–21, and dexamethasone is administered weekly. The treatment duration is 2 years (24 cycles). Patients eligible for autologous transplantation undergo stem cell collection after 6 cycles of therapy. The primary endpoint is the rate of sustained minimal residual disease (MRD) negativity at 2 years. Preliminary analysis has demonstrated that D-RVD is well tolerated in high-risk SMM, showing significant early activity. Responses are observed to deepen over time, with patients achieving MRD-negative disease. The most common grade 3 TEAEs included neutropenia, ALT increased, thrombocytopenia, syncope hyperglycemia, hypertension, and diarrhea. No patients discontinued therapy due to toxicity [71].

Another phase 2 trial (ASCENT, NCT03289299) is evaluating an even more intense treatment regimen involving daratumumab, carfilzomib, lenalidomide, and dexamethasone (D-Krd) in patients with high-risk SMM with a potential curative intent. The primary endpoint is the rate of stringent CR, and secondary objectives include MRD negativity and progression-free survival (PFS). Treatment consists of three phases: induction (6 cycles), consolidation (6 cycles), and maintenance with lenalidomide (12 cycles). Each cycle is 28 days. The best overall response rate was 97% (37% sCR, 26% CR, 29% VGPR). 84% of patients became marrow MRD negative. PFS rate at 3 years was 89.9%. D-Krd given for a fixed duration of 2 years was associated with high response rates and deep responses, including high rates of MRD negativity. Any grade toxicity, possibly related to therapy, was observed in 97% of patients. Grade ≥ 3 hematological toxicity in 15% of patients and non-hematological toxicity in 51% of patients [72,73].

A phase 2 trial (NCT02960555) is investigating single-agent isatuximab (ISA), an anti-CD38 IgG1 monoclonal antibody that targets a different epitope than daratumumab, in patients with high-risk SMM for a fixed duration of 30 cycles. Patients receive ISA IV at 20 mg/kg dose on days 1, 8, 15, and 22 (cycle 1), on days 1 and 15 (cycles 2–6) and on day 1 (cycles 7–30). Treatment repeats every 28 days. The primary endpoint is the overall response rate (ORR). Outcomes and response rates were similar to that seen with lenalidomide (ORR of 62.5%, including a VGPR or better rate of 22%). There were five grade 3 TEAEs that resolved to baseline (dyspnea related to infusion reaction, headache, ANC decrease, urinary tract infection). Most common grade 1–2 related adverse events: nausea, vomiting, WBC decrease, diarrhea, fatigue, headache, mucositis, myalgia and infusion reaction. This study also evaluated the quality of life, suggesting that ISA, by the end of cycle six of treatment, reduces anxiety and concern about progression to MM [74].

A Phase 3 randomized trial ITHACA (NCT04270409) evaluated isatuximab in combination with lenalidomide and dexamethasone compared to lenalidomide and dexamethasone in patients with high-risk SMM Enrolled patients received ISA 10 mg/Kg IV on day 1, 8, 15, and 22 in cycle 1, on day 1 and day 15 cycles 2–12, on day one in cycle 13–36; plus lenalidomide days 1–21 (25 mg 1–9 cycles; 10 mg in 10–24 cycles) and dexamethasone weekly. The cycle duration was 28 days. An initial safety run-in analysis has shown a safety profile and encouraging results, with ORR of 100% at a median follow-up of 19.4 months (sCR/CR in 43.5% and ≥VGPR in 73.9% of patients). Grade ≥ 3 TEAEs were reported in 47.8% of patients (COVID-19 pneumonia, insomnia, pneumonia, hyperglycemia, agitation, lethargy, gastroesophageal reflux disease, retinal detachment, papular rash, and muscle spasms). Grade 3–4 neutropenia occurred in 30% of patients, and grade 3 thrombocytopenia in 4% [75].

#### 6.1.2. Anti-SLAM F7/CS1 Monoclonal Antibodies

Researchers are exploring the activity of Elotuzumab (Elo), a monoclonal antibody anti-SLAM F7/CS1 in SMM.

Elo stimulates antibody-dependent cellular cytotoxicity (ADCC) by engaging with CD16 on NK cells and antibody-dependent cellular phagocytosis (ADCP) by macrophages [81]. Interestingly, SLAMF7 is also expressed in cytolytic NK cells. Although in relapsed/refractory MM, Elo has not demonstrated efficacy as a single agent, it is recommended in combination with lenalidomide or bortezomib, therapy that enhances anti-tumor responses by NK cells [82]. Moreover, Elo may be more effective in first-line therapy when the immune system is still intact.

In high-risk SMM, Elo has been studied alone and in combination with other agents.

A phase 2 study [76] (NT01441973) evaluated Elo monotherapy at two different doses: 10 mg/kg, weekly cycles 1 and 2 and every 2 weeks after that and 20 mg/kg, days 1–8 cycle 1, then monthly in 31 patients with high-risk SMM. The primary endpoint was the correlation between the reduction in monoclonal protein and the proportion of BM-derived CD56dim NK cells; secondary endpoints included ORR and PFS. However, no relationship was found between baseline CD56dim NK cells and response. With a median follow-up of 28 months, the ORR was 10%, while the 2-year PFS was 69%. Elotuzumab monotherapy was generally well tolerated, with a safety profile consistent with prior elotuzumab studies; the upper respiratory tract infections were the most common adverse events (58%), and only one was grade 3–4.

Elo has shown synergistic activity when combined with lenalidomide and dexamethasone in relapsed/refractory MM [82], potentially making it a better alternative for SMM. Elotuzumab, lenalidomide, and dexamethasone were tested in a phase 2 randomized study (E-PRISM, NCT02279394) in high-risk SMM. Fifty patients were enrolled [77]. The primary endpoint was progression-free at 2 years. The study’s primary analysis showed a CR rate of 6%, a VGPR rate of 37%, and 84% of the patients achieved at least partial remission (PR). No differences in response were found in patients with high cytogenetic alterations [5]. The median PFS and OS were not attained, and at 3 years, no progressions to MM were observed. The most common grade 3 or more adverse events were hypophosphatemia (34%), neutropenia (26%), and lymphocyte count decreased (22%). No significant toxicities emerged.

#### 6.1.3. Anti PD-1 Monoclonal Antibodies

Pembrolizumab, an anti-PD-1 monoclonal antibody and a checkpoint inhibitor, exhibits in vitro immunologic activities in MM [83]. A pilot early phase I trial (NCT02603887) aimed to evaluate pembrolizumab’s efficacy and safety in 13 patients with intermediate/high-risk SMM. It was administered at 200 mg IV every 21 days for up to 8 cycles. In case of clinical benefit, patients could continue up to 24 cycles [78]. After a median of eight cycles, one patient with 17p deletion and high-risk gene-expression signature reached sCR with bone marrow minimal residual disease (MRD) negativity. Eleven patients had a stable disease, and one patient progressed. Due to immune-related adverse events, three patients had to interrupt the treatment. Three patients discontinued the treatment due to transaminitis and nephritis, respectively, which were considered immune-related adverse events.

Transcriptome sequencing performed on bone marrow at baseline highlighted that patients who responded to pembrolizumab had an increased interferon-γ activity. In contrast, non-responsive patients had an increase in T cell exhaustion. The patient’s immunological profile could be a marker to predict the effectiveness of the therapy. Moreover, trials with checkpoint inhibitors in active MM have been halted due to safety concerns [84], thus limiting the scientific community’s interest in their use in precursor conditions.

#### 6.1.4. Anti-Interleukin-6 Monoclonal Antibody

A randomized, double-blind, placebo-controlled study (NCT01484275) evaluated siltuximab, an IL-6 blocking antibody, in 85 high-risk SMM. Interleukin-6 (IL-6) promotes myeloma cell growth and survival, inhibiting apoptosis [85]. Patients received siltuximab (15 mg/kg IV every four weeks until progression or toxicity) or placebo [79]. The primary endpoint of the study was one-year PFS, which resulted in 84.5% in the experimental arm compared to 74.4% in the placebo arm at a median follow-up of 29.2 months. The median PFS with siltuximab was not reached, whereas it was 23.5 months with placebo. Adverse events in the experimental arm were mainly infections and urinary complications. Although the main endpoints have not been fully met, siltuximab’s role in preventing SMM progression needs to be further studied.

### 6.2. Monoclonal Antibodies in MGUS and SMM Low Risk

#### Anti-CD-38 Monoclonal Antibodies

The treatment of patients with MGUS and low-risk SMM is complex. It requires effective and safe treatments in patients who have a low risk of evolving into symptomatic disease. Furthermore, in these trials, evaluating a benefit in terms of OS requires a very long time.

The efficacy of the anti-CD38 monoclonal antibody in delaying progression to multiple myeloma was assessed in high-risk MGUS or low-risk SMM patients in the phase 2 study, D-PRISM (Precision Intervention Smoldering Myeloma, NCT03236428). Daratumumab is administered IV weekly during cycles 1–2, every other week during cycles 3–6, and monthly during cycles 7–20. The primary endpoint is the achievement of at least VGPR after 20 cycles [86]. Thirty-one patients, including two patients with high-risk MGUS, have been enrolled. Dara was very well tolerated among patients. At a preliminary analysis 15 patients completed a median of six cycles; partial response occurred in 73% and VGPR in 20% of these patients. No patient has progressed to MM or discontinued therapy. Grade 3 toxicities were rare, occurring in only 2 out of 28 patients, and included diarrhea and flu-like symptoms. The most common toxicities of any grade included fatigue, cough, nasal congestion, headache, hypertension, nausea, and decreased white blood cell count. No patients have discontinued therapy due to toxicity. 

### 6.3. Monoclonal Antibodies in MGRS

#### Anti-CD-38 Monoclonal Antibodies

A related question involves the efficacy of pharmacological intervention in MGRS. Several clinical trials are currently assessing the impact of anti-plasma cell therapy with anti-CD38 monoclonal antibodies on renal outcomes in patients with MGRS. A retrospective analysis reported 25 MGRS patients treated with daratumumab-based therapy: 77% of patients achieved a hematologic response, and 55% of patients showed a >30% reduction in proteinuria at 6 months from the start of therapy with stable glomerular filtration rate (eGFR). The toxicity was mild and predictable [87].

A phase II trial (NCT03095118) assessed the efficacy and safety of daratumumab in patients with MGRS associated with proliferative glomerulonephritis with monoclonal immune deposits (PGNMID) and C3 glomerulopathy (C3G). They received daratumumab IV (16 mg/kg) once weekly for eight weeks and then every other week for eight doses. The primary endpoint was safety; secondary endpoints included CR and PR. CR was defined as proteinuria < 500 mg/d with <15% decline in baseline eGFR; PR was defined as >50% reduction in 24-h proteinuria with <30% decline in eGFR. Over 12 months, six of the ten patients with PGNMID had a PR, and four had a CR. Daratumumab showed an acceptable safety profile and significantly improved proteinuria while stabilizing kidney function. These preliminary results are favorable and should be further investigated [88].

Currently, there is an ongoing phase II single-arm trial (NCT04614558) investigating the role of isatuximab in improving kidney function in patients with MGRS. Isatuximab is administered IV at a dose of 10 mg/kg weekly for four doses and every other week for a total of 6 months.

Another active phase II trial (NCT06083922) is currently recruiting participants to evaluate the combination of cyclophosphamide, bortezomib, dexamethasone with daratumumab SC (CyBorD-Dara) for the treatment of MGRS and cast nephropathy. The regimen involves eight cycles of CyBorD-Dara. Patients eligible for autologous stem cell transplantation (ASCT) undergo ASCT after completing eight cycles of induction. For patients ineligible for ASCT, the recommendation is to continue maintenance therapy with daratumumab SC administered every 4 weeks, bortezomib, and dexamethasone administered every other week for 2 years from the start of treatment. Researchers aim to determine whether this combination is an effective treatment for these conditions.

## 7. Discussion

Monoclonal antibodies have profoundly reshaped the treatment landscape of various hematologic malignancies, including multiple myeloma. Despite the therapeutic advances, multiple myeloma persists as an incurable disease.

In recent years, significant progress has been made in understanding the pathogenesis of this disease, and new therapies, such as immunomodulatory agents and MoAbs, have been introduced that are safe and effective. The research focused on how to reactivate the altered immune system and to prevent the progression of precursor conditions, MGUS and SMM., towards active disease [63,89,90,91].

As we have seen before, the first class of drug that was tested in SMM was lenalidomide, either alone or with dexamethasone. Two pivotal trials [7,8] showed a significant improvement in time to progression and prevention of organ damage in high-risk SMM, but only one of these studies indicated an overall survival benefit [60].

However, the impact of these studies on clinical practice is burdened by several limitations. First, only one study has OS as the primary endpoint. Additionally, the case series are small in size and have short follow-up periods. Moreover, patients included in these studies may have already met the criteria for reclassification as symptomatic myeloma according to the new IMWG 2014 criteria [5]. These considerations make the interpretation of results more challenging.

These results remain the cornerstone on which subsequent phase 2 and phase 3 studies with different pharmacological agents are built to optimize the results obtained from lenalidomide. Ongoing trials examine new therapies, such as MoAbs alone or in combination regimens incorporating other targeted agents, such as immunomodulatory drugs and proteasome inhibitors.

In an attempt to intervene during the early stages of the disease, phase II is assessing the use of the MoAb, Daratumumab, as a single agent in patients with high-risk MGUS (NCT03236428). However, current data are inadequate to support intervention in the MGUS context, and an active observation approach is presently recommended. Regarding MGRS, the use of MOAbs is proving promising in preserving organ function, however the data are still preliminary.

Early treatment has raised some critical considerations that clinical trials must address.

The safety profile of early treatment in MGUS and SMM is a critical consideration since these patients are asymptomatic compared to those with MM. It is essential to carefully monitor the side effects induced by both short- and long-term therapy. These side effects can potentially have a lasting impact on their quality of life and may be associated with poor compliance and a higher attrition rate in clinical trials. Emerging evidence from ongoing studies suggests that lenalidomide and monoclonal antibodies, whether used as single agents or in combination, demonstrate a low toxicity profile in the early treatment of MGUS and SMM.

Moreover, it is essential to evaluate the risk of secondary tumors associated with early therapy. In MM, the association between the disease and SPMs as a long-term risk has long been recognized. However, many studies in MM have concluded that the benefits of therapy outweigh the risks of developing SPMs. The risk of SPMs is influenced by various factors related to the patient, the disease, and the therapy. Immunomodulatory drugs, specifically lenalidomide, have been associated with an increased incidence of SMPs in newly diagnosed, relapsed, and refractory patients. Newer anti-myeloma therapy, such as proteasome inhibitors and monoclonal antibodies, did not appear to increase SPM risk, although long-term follow-up data are lacking [92,93,94]. In the contest of SMM, no alarming signals have emerged thus far, but the available data are still preliminary and insufficient to draw long-term conclusions. Therefore, clinical trials evaluating emerging anti-myeloma therapy should be designed to include enhanced monitoring, precise measurements, and well-defined endpoints for secondary cancers, especially in SMM.

Furthermore, there is a concern that early intervention in MGUS and SMM may select clones that are resistant to future therapies and could have a mutagenic effect on the clonal cells [95].

A crucial point in MGUS and SMM is to determine how to treat these patients and what intensity and type of treatment is preferable. The first possibility is that of a preventive approach; the therapy allows the immune system to be reactivated to control the growth of the pathological clone; the second possibility is that with a curative purpose, using an intensive therapy that combines multiple drugs but with the risk of developing more significant toxicities related to treatment, to eradicate the pathological clone. The optimal therapeutic approach, whether “curative” or “preventive”, remains under investigation [9].

Identifying patients with SMM and MGUS who are at the highest risk for progression is one of the greatest challenges. Although great strides have been made in recent years in the creation of risk stratification models, we still do not have adequate clinical, laboratory, and biological parameters that allow us to identify patients who could best benefit from early therapeutic intervention [25,63,96]. Furthermore, to date, clinical studies use discordant risk classification criteria. These considerations make comparison and interpretation of results difficult and make it urgent to standardize these criteria.

Furthermore, given the increasing knowledge of how immune dysfunction affects SMM evolution and the possible effects of therapies on immunity, state-of-the-art evaluation of patient immune status and appearance/increase of anti-cancer immunity following treatment might further improve our understanding of the effects of treatment of SMM. Similarly, optimal appropriate patient selection for clinical trials should include not only disease-intrinsic risk factors but also evaluation of anti-MM immunity to identify subsets of patients with features that could predict higher benefit, or higher need, of specific immunomodulatory agents.

Another issue concerns the endpoints used in clinical trials in these precancerous conditions. The primary endpoint should be an OS benefit, but this takes a long time, especially in patients who may take a long time to progress. Therefore, ongoing clinical trials more commonly use several endpoints as surrogates for OS, such as PFS and depth of response to therapy. PFS may represent a lead-time bias only in these patients, and the quality of response to treatment may not be as relevant as in that of active disease patients.

Other endpoints that may be clinically relevant in clinical trials are sustained MRD negativity, PFS2, defined as the time from randomization to progression to next-line treatment or death, or the onset of end-organ damage such as bone disease symptomatic or renal failure or the occurrence of health-related quality of life (QOL) events. Even in this sector, harmonization of the primary and secondary endpoints in the different studies would be fundamental [97].

Moving to earlier therapy in SMM and MGUS has several financial implications. While early treatment has the potential to improve patient outcomes and quality of life by preventing end-organ damage and reducing the burden of disease progression, it also brings significant costs that need to be weighed against the potential benefits. Therefore, a thorough economic evaluation, including cost-effectiveness analysis and long-term financial modeling, is essential to guide policy decisions and ensure sustainable healthcare practices.

In conclusion, there is an urgent need to delineate the genomic landscape of MGUS and SMM and to enhance risk stratification models in asymptomatic disease. Simultaneously, efforts to optimize early treatment for asymptomatic disease are crucial. The clinical benefit of early therapy in high-risk SMM shows promise. Results from ongoing trials of innovative treatment combinations and novel therapeutic approaches will offer clarity and are poised to transform the treatment landscape shortly. The preliminary results appear promising, though an adequate follow-up is needed to detect the potential benefit, especially for OS.

## Figures and Tables

**Table 1:** Risk stratification models to predict MGUS progression to MM.

Model	Risk Factors	Outcomes
Mayo (2005) [27]	1. MC > 15 g/L 2. Non Ig G subtype 3. Abnormal FLC ratio	PFS at 20 years: 0 risk factors: 5% 1 risk factor: 21% 2 risk factors: 37% 3 risk factors: 58%
Swedish study (2014) [28]	1. MC > 15 g/L 2. Non Ig G subtype 3. Abnormal FLC ratio 4. Immunoparesis (reduction of ≥1 uninvolved heavy chain)	PFS at 10 years: 0 risk factors: 4% 1 risk factor: 6% 2 risk factors: 12% 3 risk factors: 23% 4 risk factors: 40%
PETHEMA (2007) [29]	1. Aberrant phenotype in >95% of BMPCs 2. DNA aneuploidy	PFS at 5 years: 0 risk factors: 4% 1 risk factor: 46% 2 risk factors: 72%
PETHEMA (2010) [30]	1. Aberrant phenotype in >95% of BMPCs 2. Evolving MGUS (>10% increase in M-protein by the third year as confirmed by two consecutive measurements separated by ≥1 month)	PFS at 7 years: 0 risk factors: 2% 1 risk factor: 15% 2 risk factors: 72%

Abbreviations. BMPC: clonal bone marrow plasma cells; FLC: free light chain; MC: monoclonal component.

**Table 2:** Risk stratification models of progression from SMM to active MM.

Model	Risk Factors	Risk Groups	Outcomes
PETHEMA (2007) [29]	1. ≥95% aberrant BMPCs by flow cytometry (defined as CD38^+^ cells with absence or underexpression of CD19 and/or CD45 or overexpression of CD56) 2. Immunoparesis (reduction of ≥1 uninvolved heavy chain)	0, low risk 1, intermediate 2, high risk	PFS at 5 y: Low risk, 4% Intermediate risk, 46% High risk, 72%
Mayo (2008) [31]	1. BMPCs ≥ 10% 2. MC ≥ 30 g/L 3. sFLC ratio ≤ 0.125 or ≥8	0/1, low risk 2, intermediate risk 3, high risk	PFS at 5 y: Low risk, 25% Intermediate risk, 51% High risk, 76%
SWOG (2014) [32]	1. MC > 30 g/L, 2. Involved sFLC > 25 mg/dL, 3. GEP-70 > 0.26	0, Low 1, Intermediate (1 factor) ≥2 factors, High	PFS at 52 y: Low risk, 9.7% Intermediate risk, 26.3% High risk, 47.4%
Mayo 20/20/2 (2018) [33]	1. BMPCs > 20% 2. MC > 20 g/L 3. sFLC ratio < 0.05 or >20	0, low risk 1, intermediate risk 2–3, high risk	Median TTP: Low risk, 110 mo Intermediate risk, 68 mo High risk, 29 mo
IMWG (2020) [34]	1. sFLC ratio: 0–10, 0 points; 10–25, 2 points; 25–40, 3 points; >40, 5 points 2. MC (g/L): 0–1.5, 0 points; 1.5–3, 3 points; >3, 4 points 3. Percentage of BMPCs 0–15, 0 points; 15–20, 2 points; 20–30, 3 points; 30–40, 5 points; >40, 6 points 4. FISH abnormalities * No, 0 points; Yes, 2 points	0–4 points, low risk 5–8 points, low/intermediate risk 9–12 points, intermediate risk >12 points, high risk	Risk of progression at 2 y: Low risk, 3.8% Low/intermediate risk, 51.1% Intermediate risk, 26.2% High risk, 72.5%

* FISH abnormalities include t(4;14), t(14;16), 1q gain, and del 13q/monosomy 13. Abbreviations. GEP-70: University of Arkansas Medical Sciences 70-gene expression profile signature; TTP: time to progression; MC: monoclonal component; sFLC: serum free light chains; BMPC: bone marrow plasma cells; PFS: progression-free survival; mo: month

**Table 3:** Monoclonal antibodies in high-risk SMM: selected clinical trials.

Study	Phase N pts	High-Risk Definition	Design	Control Arm	Primary Endpoint	Response Rates	Survival Outcomes
CENTAURUS/ NCT02316106 [67,69]	2, Randomized N = 123	BMPCs ≥ 10% and at least 1 of the following: MC ≥ 30 g/L (IgA ≥ 20 g/L), urine M protein > 500 mg per 24 h, abnormal sFLC ratio (<0.126 or >8),	Daratumumab 16 mg/kg IV in 8-wk cycles (C): Extended intense: C1 every 1 wk; C2–3 every other wk; C4–7 every 4 wks; C8–20 every 8 weeks. Intermediate intense: C1 every 1 wk and C2–20 every 8 weeks Short dosing: C1 every 1 wk	Observation	CR	CR at 15.8 months: 2.4% vs. 4.9% vs. 0% CR at 25.9 months: 4.9% vs. 9.8% vs. 0% CR at 84 months: 4.9% vs. 12.2% vs. 0%	PFS at 2 years: 89.9% intense vs. 82.0% intermediate vs. 75.3% short OS at 84 months: 81.3% intense vs. 89.5% vs. 88.1% short
AQUILA/ NCT03301220 [70]	3, randomized N = 390	BMPCs ≥ 10% and ≥1 of the following: MC ≥30 g/L, IgA SMM, immunoparesis, abnormal sFLC ratio ≥8 to < 100, or BMPCs > 50% to <60%	Daratumumab SC: C1–2, every 2 weeks in C3–6, and every 4 weeks thereafter until C39 (28 days/cycle), up to 36 months, or until disease progression	Observation	PFS	NA	NA
DETER-SMM/ NCT03937635	3, randomized N = 288	The presence of 2 or more of the following factors: abnormal sFLC ratio (>20, or <100); MC ≥ 20 g/L; high-risk FISH; BMPCs > 20%	Daratumumab 16 mg/kg IV on days 1, 8, 15, and 22 of C1–2, days 1 and 15 of C3–6, and day 1 of C7–24. Lena daily on days 1–21. Dex on days 1, 8, 15, and 22 in C1–12. (28 days/cycle) up to 24 courses or until disease progression	Rd	OS	NA	NA
B-PRISM, NCT04775550 [71]	2, single arm N = 60	BMPCs ≥ 10% and any one or more of the following: MC ≥ 30 g/L; immunoparesis; sFLC ratio (≥8<100); progressive increase in M protein level; BMPCs 50–60%; abnormal plasma cell immunophenotype; high-risk FISH; focal bone lesion or high risk according to IMWG/Mayo 2018 “20-2-20” Criteria (at least 2 of the following)	Daratumumab SC standard dose and schedule, bortezomib given weekly on days 1, 8, and 15 for C1–6 and then biweekly until completion of C24. Lena on days 1–21 and Dex weekly. Up to C24.	No	MRD negativity at 2 years	NA	NA
ASCENT, NCT03289299 [72,73]	2, single arm N = 46	The presence of any 2 of the following: MC > 20 g/L; sFLC ratio > 20; BMPC > 20%; IMWG score ≥ 9 using risk scoring system using sFLC ratio, MC, marrow plasma cell percentage, and presence of high-risk FISH	Daratumumab 16 mg/kg IV plus KRd standard dose and schedule. Induction: C1–6 of Dara-KRd Consolidation: C7–12 of consolidation with Dara-KRd Maintenance: C12–24 with Dara and Lena.	No	sCR	ORR 97%, 37% sCR, 26% CR, 29% VGPR, 2% PR, 1% SD 84% patients MRDneg 61% also in CR	PFS at 3 years 89.9%
NCT02960555 [74]	2, single arm N = 61	NA	Isatuximab 20 mg/kg IV (28 days/cycle), C1 every 1 wk; C2–6 every other wk; C7–30 every 4 weeks	no	ORR	ORR 64%, CR 5%, with MRD negativity	NA
ITHACA/ NCT04270409 [75]	3, randomized N = 337	The Mayo ‘20-20-20′ and/or PETHEMA model criteria	Isatuximab 10 mg/kg IV on day 1, 8, 15, and 22 C1, day 1 and 15 C2–12, day 1 C13–36; plus Lena D1–21 (25 mg C1–9; 10 mg C10–24) and Dex weekly (40 mg, 20 mg for ≥75 yr-old pts C1–9; 20 mg C10–24). 28 days/cycle.	Rd	PFS	ORR of 100% (median follow-up of 19.4 months): 13.0% (sCR), 30.4% CR 30.4% VGPR	NA
NT01441973 [76]	2, single arm N = 41	The presence of MC ≥ 30 g/L with BMPC ≥ 10%; or MC 10–30 g/L (alternatively urine M protein > 200 mg/24 h), BMPCs ≥ 10% and sFLC ratio < 0.125 or >8.0	Elotuzumab monotherapy: 20 mg/kg (days 1 and 8 C1, monthly from C2) 10 mg/kg (weekly cycles 1 and 2, twice monthly from C3)	No	The association between NK cell status and M protein reduction	ORR: 10%	PFS at 2 years: 69%
NCT02279394 [77]	2, single arm N = 51	Mayo/or PETHEMA criteria	Induction: 28 day-cycle C1–2 Elotuzumab 10 mg/kg IV. days 1, 8, 15, 22 + Lena 25 mg days 1–21 + Dex 40 mg days 1, 8, 15, 22 C3–8: Stem cell collection; Elotuzumab 10 mg/kg IV days 1, 15 + Lena 25 mg days 1–21 + Dex 40 mg days 1, 8, 15 Maintenance: 28 day-cycle (C9–24) Elotuzumab 10 mg/kg IV. days 1 + Lena 25 mg days 1–21	no	PFS at 2 years	ORR: 84% VGPR: 37% CR: 6%	None of the patients progressed to MM at 3 years
NCT02603887 [78]	Pilot study, single arm N = 13	PETHEMA, Mayo 2008 or SWOG criteria	Pembrolizumab 200 mg IV every (21 days/cycle) × 8 cycles, with an option to continue up to 24 cycles if continued benefit.	no	ORR	ORR: 8%, CR: 8%, MRD negativity 8%	15% of patients progressed to MM
NCT01484275 [79]	Pilot study, randomized N = 85	BMPCs > 10% and either MC > 3 g/dL, or sFLC ratio < 0.126/>8 and MC > 10/<30 g/L	Siltuximab 15 mg/kg IV (28 days/cycle) until progressive disease.	Observation	PFS	NA	1-year PFS: 84.5% vs. 74.4% (*p* < 0.06) Median PFS: NR vs. 23.5 months; OS NR in both arms

Abbreviations: SMM: smoldering multiple myeloma; Dara: Daratumumab; Lena: lenalidomide; Rd: lenalidomide dexamethasone; Dex: dexamethasone; Dara-KRd: Daratumumab–carfilzomib–lenalidomide–dexamethasone; BMPC: clonal bone marrow plasma cells; sFLC: serum free light chain; MC: monoclonal component; pts: number of patients; CR: complete response; sCR: stringent complete response; M: monoclonal; VGPR: very good partial response; MRD: minimal residual disease; PFS: progression-free survival; OS: overall survival; wk: week; C: cycle; FISH: Fluorescence In Situ Hybridization; ORR: overall response rate; NA: not applicable.

## Data Availability

All data presented derive from published papers.
